# Cancer risk management in Colombia, 2016

**DOI:** 10.25100/cm.v49i1.3882

**Published:** 2018-03-30

**Authors:** Paula Ramírez-Barbosa, Lizbeth Acuña Merchán

**Affiliations:** Cuenta de Alto Costo- Fondo Colombiano de Enfermedades de Alto Costo. Ministerio de Salud y Proteccion Social, Bogota, Colombia

**Keywords:** cancer, health records, quality indicators, health care, cáncer, registros de salud, indicadores de calidad

## Abstract

**Objective:**

To describe the outcomes of risk management indicators for five types of cancer in population that is affiliated to the General System of Social Security in Health, in six cities of Colombia.

**Methods:**

Based on the data from the administrative cancer registry for the period 2016, the High Cost Disease Fund (CAC in Spanish) as a technical organization of the Colombian health system, processed and analyzed the data for the calculation of risk management indicators established in consensus based on the evidence found in six cities

**Results:**

There is a diversity in the indicators results found among the different cities, evidencing strengths and weaknesses in each of them for the different types of cancer. From the set of indicators, those with the best results presented are related to the greater detection of cancer in early stages or in situ, as well as a decrease in mortality, especially in colorectal and in gastric cancer. Most indicators in gastric cancer showed optimal results. Important measurements such as the opportunity for diagnosis and treatment are below the proposed standard for most types in all the six cities.

**Conclusions:**

The descriptive analysis of cancer risk management indicators shows certain weaknesses in the quality and timeliness of the care of cancer patients, the standards agreed upon in the consensus with the different actors of the system are not being reached, situation which may be due to a reality of problems of the Colombian health system, as well as deficiencies in the quality of the report to the CAC.

## Resumen

**Objetivo:**

Describir los resultados de los indicadores de gestión del riesgo para cinco cánceres en la población afiliada al Sistema General de Seguridad Social en Salud en Colombia en ciudades que tienen registros de cáncer.

**Metodos:**

La Cuenta de Alto Costo (CAC) procesó y analizó la información del registro administrativo nacional de cáncer del periodo 2016 en Cali, Pasto, Bucaramanga, Manizales, Barranquilla y Medellín; para calcular los indicadores de gestión del riesgo establecidos en el consenso basado en la evidencia para la atención en cáncer.

**Resultados:**

Existe diversidad en los resultados de los indicadores entre las diferentes ciudades, evidenciando fortalezas y debilidades en cada una de ellas y para los diferentes tipos de cáncer. Los indicadores que mejores resultados presentaron se relacionan con la mayor detección del cáncer en estadios tempranos, así como disminución de la mortalidad, especialmente en cáncer de colorrectal y en cáncer gástrico. El cáncer gástrico fue aquel que mayor cantidad de indicadores obtuvieron resultados óptimos. Mediciones importantes como la oportunidad de diagnóstico y de tratamiento se encuentran por debajo del estándar propuesto para la mayoría de los tipos de cáncer y de las ciudades.

**Conclusiones:**

Se evidencia ciertas debilidades en la calidad y la oportunidad de la atención oncológica, no se están cumpliendo los estándares acordados en los consensos con los diferentes actores del sistema: puede deberse a una realidad de problemas del sistema de salud colombiano, así como a la necesidad de fortalecer la calidad del reporte por parte de las entidades a la CAC.

## Introduction

Cancer is a public health problem in Colombia and the world, which requires decisive interventions to confront and contain it. For this reason, different strategies and policies have been developed in different countries and must be based not only on clinical but also on epidemiological knowledge of cancer, which must also be tied to the administrative actions that are carried out at the health insurance level. In order to accomplish this, the way to know and monitor these actions is through the measurement of indicators that account for the quality of health care ^1^ ^,^ ^2^.

In Colombia, information related to cancer has taken great importance over the years and the country works to obtain sufficient, real and accurate information with the objective of analyzing and addressing it to the processes, evidencing strengths and weaknesses within the health system for the formulation of strategies, programs and policies that define corrective interventions. To achieve this purpose, the Ministry of Health and Social Protection of Colombia (MSPS in Spanish) through Resolution 4496 of 2012 organizes the National Cancer Information System (SINCan) ^3^, in which the High Cost Diseases Fund (CAC in spanish) is part of these information sources, analyzing data related to insurance and the provision of services to cancer patients in the country. Similarly, the MSPS-Colombia with Resolution 0247 of 2014, establishes the report for the registration of cancer patients where the High Cost Diseases Fund is responsible for collecting and consolidating the information that the healthcare payer, including those of the exception regime and the public institutions, private, and mixed health service providers, as well as the departmental, district and municipal health authorities, are mandated to report ^4^.

Therefore, Colombia has a national administrative registry in cancer (RANC in Spanish) since 2014, with clinical, administrative, sociodemographic and cost components which, since 2015, have been audited to guarantee the quality of the information as a complement to other sources of information. Based on this information, in 2016 the High Cost Diseases Fund began the construction and development of processes to standardize measurements in the cancer care process through consensus based on evidence for the formulation of indicators that measure the management conducted by insurers and providers on people with cancer in the country.

Likewise, another source of information is population-based cancer registries (RCBP in Spanish) in six cities of the country that collect and classify new cases of cancer in permanent residents of Cali, Pasto, Bucaramanga, Manizales, Barranquilla and Medellin. They are members of the International Association of Cancer Registries (IACR) and have disseminated information on incidence and survival in *Cancer Incidence in Five Continents* ^5^ and in the CONCORD study ^6^ . The objective of this paper is to describe the results of risk management indicators for five types of cancer (stomach, colorectal, breast, cervix and prostate) in the population that is affiliated with the Colombian General System of Social Security in Health (SGSSS in Spanish) in the six cities of Colombia that have RCBP.

## Materials and Methods

The High Cost Diseases Fund, is a technical body of the General System of Social Security in Health of Colombia with the mission of Promoting risk management, the generation of health outcomes and knowledge management, through the articulation of different SGSSS actors to decrease the trend of High Cost events, stabilize the variability in their management, ensure technical-scientific quality and reduce the impact of the current disease burden, through various mechanisms. The insurers and health providers are mandated to report the data of all cancer patients to the CAC on an annual basis. The CAC is a source of information of the SINCan responsible for integrating the information to form the National Administrative Registry of Cancer (RANC)

### SINCan 

**Figure 1:**
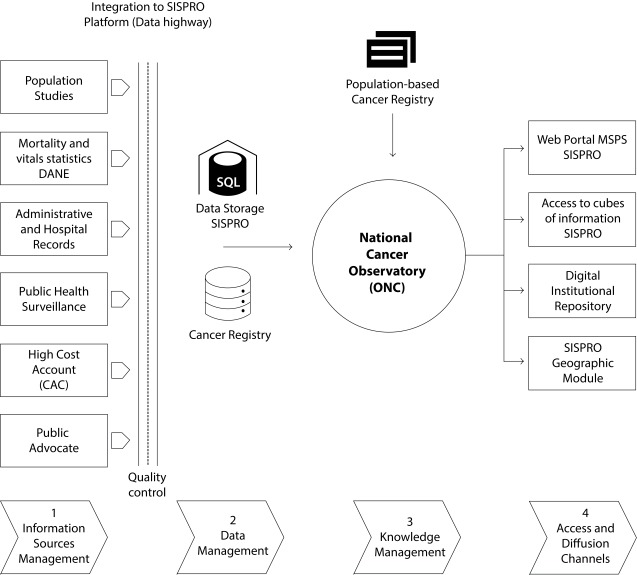
National Cancer Information System of Colombia

The available sources within the SINCan are administrative and hospital records; as well as population studies and surveys. The data reported and collected from the local, territorial and national level are integrated into a data warehouse that allows interoperability of the sources, which is called the Social Protection Comprehensive Information System (SISPRO) (Fig. 1). The National Cancer Observatory consolidates the information of the SISPRO, RANC and Cancer Population Registries in order to build the indicators to monitor the situation in the country, the analysis plans, and the information outputs as necessary tools to adequately manage knowledge about mortality, morbidity, access to services and actions for cancer control in Colombia. 

### Case definition

People with histopathological diagnosis of some type of *in situ* or invasive cancer; people with clinical diagnosis of cancer, supported and justified in clinical, biochemical, imaging or laboratory tests appropriate in those for whom, due to any clinical condition negative to its performance or contraindication, it was not possible to perform histopathological confirmation until the cutoff date, but who are being managed as cancer patients. For specific cancers, codes of the International Classification of Diseases, Tenth Revision (ICD-10) were used as follows: stomach (C16); colorectal (C18 to C21); breast (C50); Cervix (C53) and prostate (C61).

### Collection instrument

Structured form that collects information in the following aspects: General identification of the health insurer and the reported user (Questions (Q) 1-16); General information related to diagnosis, staging and treatment objectives (Q 17-41); antecedents that precede the diagnosis of the cancer reported (Q 42-73); specific surgery information (Q 74-85); radiotherapy specific information (Q 86-105); specific information on hematopoietic stem cell transplantation (Q 106-110); specific information on complementary treatment (Q 111-124); current situation of the user at the cut-off date (Q 125-132)

### Information quality control in the High Cost Disease Fund (CAC)

The CAC audits the information given by the EAPB against the clinical records. Its objective is to verify the authenticity of the data reported and to be able to accurately conclude the available information. This process consists of two main components: the first refers to the audit carried out by the information system; through a validation mesh and the second, of the information against the medical record.

### Process of creating the CAC indicators

The development of the consensus done among the different actors of the General System of Social Security in Health in Colombia for the identification of indicators in cancer arose from the need to evaluate and monitor risk management in cancer carried out by insurance entities and health service providers, in order to be aligned and contribute to the strategies that the country has implemented for cancer control, such as the 10-year plan for cancer control in Colombia ^1^, clinical practice guidelines, and comprehensive care routes for different types of cancer; this way generating control mechanisms and providing a guide to health professionals, insurance entities, providers and other actors in the search for a better risk management in health that contributes to decrease clinical variability, reduce the complications of the disease, improve survival and the quality of life of patients, and control spending on health.

The consensus has been developed by the High Cost Diseases Fund since 2016, giving priority to the most prevalent types of cancer in Colombian society, and according to the priorities of the Ministry of Health; To this end, the concepts of the methodological manual of deliberation and participation of the Institute of Technological Evaluation in Health (IETS in Spanish) were adopted, and the methodology proposed by the methodological guide for the preparation of clinical practice guidelines was adapted. These guidelines present the technical processes for the formulation of the research question, the review of the literature, the grading of the scientific evidence and the process for the selection and construction of the indicators ^7^ ^,^ ^8^ . For the selection and construction of the indicators, an adaptation of the methodology "The RAND/UCLA Appropriateness Method (RAM)" was carried out ^9^, which allows combining the best available scientific evidence with the collective judgment of the experts, in this case, thematic, methodological and administrative. For the structuring of the question, the PICO strategy was taken into account ^10^ ^,^ ^11^ , which was presented for each of the types of cancer worked on and then an online application created for the virtual development of the consensus was designed, where the research question, the objectives, the scope, and the limitations were socialized and there was a space for participation for the actors involved. 

A search and critical reading of literature was carried out and it was classified according to the type of scientific evidence, using the AGREE II instrument ^12^ for the qualification of the clinical practice guidelines and the recommendations of the GRADE system ^13^ for the qualification of the review articles. Once the evidence was available, we extracted the recommendations and definitions of interest, which were reviewed and adjusted by the participants through the virtual forum, from there the possible indicators were generated (the type of these: process or result), the name, the description, the population object of application (total of cases or new cases) and the different guidelines and articles that supported the recommendation.

Finally, a group of indicators was defined and evaluated through two virtual and a third face-to-face votes. The consensus participants determined if the proposed indicators were appropriate and met three essential criteria: relevance of the indicator; feasibility, understood as the possibility of accessing the sources of information from where the data will be obtained and the validity of the content or measurement that reflects what is intended to be measured, in this case, the indicator or indicators that allow evaluating risk management in patients with cancer. Risk management indicators for different types of cancer are appended as a supplement. 

### Analysis plan

With the information reported to the CAC with a cutoff date of January 1, 2015, the baseline was calculated for each of the indicators with available information. According to the result, the cut points were defined according to the quintiles of each indicator. For the indicators without baseline, the standards were defined with the support of the clinical experts and the findings of the literature review.

The final indicators for the measurement of risk management were established with the agreement of all the participants in the third virtual meeting and the consensus was finalized. Based on this, the information is analyzed every year and weaknesses and strengths of the cancer management process are identified. 

For this occasion, the results of the risk management indicators for five types of cancer (breast (only in women), cervix, prostate, colon and rectum and gastric) in a population that is affiliated with the General Social Security System in Health will be described in six cities of the country (Barranquilla, Bucaramanga, Cali, Manizales, Medellin and Pasto) where population registries operate, as a complement to the analysis and approach of interventions for cancer control. 

The data comes from the administrative registry of cancer issued by the Ministry of Health and Social Protection (Resolution 0247 of 2014) and corresponds to the new cases reported (diagnosed) between January 2, 2015 and January 1, 2016.

We proceeded with the calculation of each of the indicators included in the evidence-based consensus designed by the High Cost Diseases Fund, which measures risk management by insurers and providers for patients with each type of cancer previously mentioned and that have defined standard cutoff points with a color for each indicator, which reflects whether the result is good (green), moderate (yellow) or bad (red).

Statistical software Stata 13 was used to process the data.

## Results

Table 1 describes the number of new cancer cases prioritized in the Ten-Year Plan for Cancer Control in Colombia that were notified to the CAC during 2015 in the six cities studied.

**Table 1:** High Cost Diseases Fund, Colombia. Number of new cancer cases for selected sites notified by health insurers during 2015.

City	Breast	Prostate	Cervix	Colon		Stomach		Total
	C50	C61	C53	C18-C21		C16		
				♂	♀	♂	♀	
Cali	235	273	59	69	80	51	42	809
Pasto	17	14	12	5	1	13	4	66
Bucaramanga	47	19	8	13	15	11	10	123
Manizales	58	29	15	13	27	20	8	170
Barranquilla	132	90	35	28	37	7	5	334
Medellin	498	250	134	103	134	80	71	1,270
Total	987	675	263	231	294	182	140	2,772

Source: Database High Cost Diseases Fund

### Breast cancer

A total of 21 indicators were included in the consensus ^14^ (Table 2), however, 19 were measured due to the availability of information in the registry. These were divided into four large groups of indicators: diagnostic and staging (indicators 1 to 7), treatment (indicators 8 to 12), opportunity (indicators 13 to 17) and results (indicators 18 to 19). It can be seen that the city of Cali had the highest proportion of patients with breast cancer who were diagnosed in situ or early stages, while Barranquilla and Pasto had the lowest proportions. Regarding the staging indicators, none of the six municipalities obtained the defined standard to consider the result as optimal, However, Medellin, for the new cases, is the one with the highest proportion of staged cases registered. The indicators related to the performance of diagnostic tests showed a low proportion of women with breast cancer and who had hormone test results, in terms of HER2 test results the proportion increased in the six cities, with Bucaramanga achieving the value considered as optimal. 

**Table 2:**
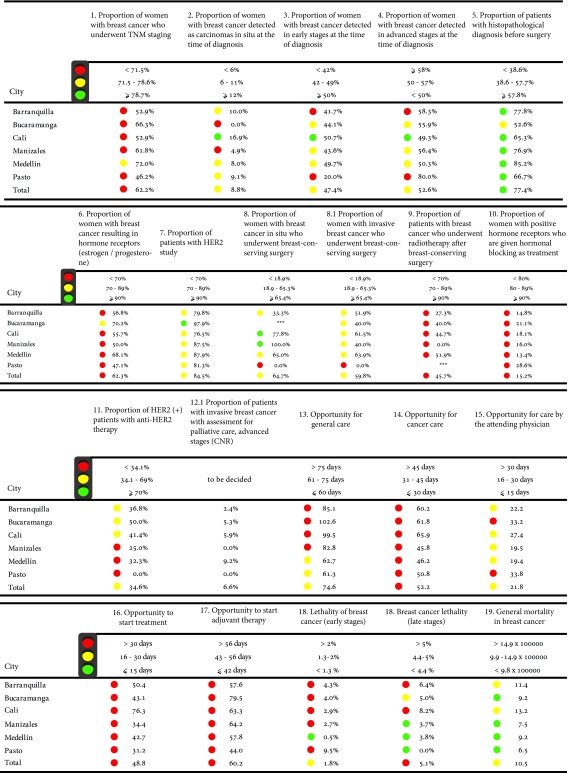
Risk management indicators in breast cancer by city of residence

Source: High Cost Diseases Fund Database Resol 0247/14 - Cut-off date: January 01, 2016

In the field of treatments such as radiotherapy, anti-Her2 therapy or administration of hormonal block, the results were located in the ranges considered as bad or moderate (red and yellow scored card) in most of the municipalities selected according to the standard of measurement of the Consensus, Cali and Medellin presented the highest proportion of patients with carcinoma *in situ* who underwent breast-conserving surgery.

In the third group of indicators, related to opportunity times, none of the cities analyzed presented a level considered optimal according to the established standards, the times for diagnosis, medical care and start of treatment presented prolonged times, above 60 days for general care, that is, from the consultation for the presence of symptoms associated with cancer up to the first treatment, however, cities such as Medellin and Pasto are close to the appropriate range. In terms of outcome indicators: mortality according to stage and mortality, the city with the best results was Medellin.

### Prostate cancer

A total of nine indicators were developed for prostate cancer ^15^ (Table 3), however 6 were susceptible to measurement, due to the absence of information in the registry.

**Table 3:**
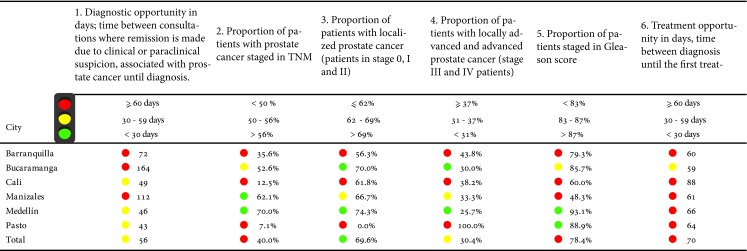
Risk management indicators in prostate cancer by city of residence

Source: High Cost Diseases Fund Database Resol 0247/14 - Cut-off date: January 01, 2016

For prostate cancer, two opportunity times were measured: from the medical suspicion for the first consultation until the diagnosis and from this until the start of the first treatment. For each one it was established that a time less than 30 days was adequate to be considered a good result. None of the cities obtained this result.

The largest proportion of patients staged with the TNM system occurred in the cities of Manizales and Medellin, and those with the highest number of patients studied in localized stages were Bucaramanga and Medellin.

The cities of Medellin and Pasto were the cities with the highest proportion of patients in the Gleason score, while the city of Manizales had the lowest proportion.

### Cervical cancer

The total number of indicators measured for cervical cancer were 12 out of a total of 14 from the consensus ^14^ (Table 4), which, like the breast cancer indicators, were divided into four large groups: diagnostic (indicator 1), treatment (indicators 2 to 6), opportunity (indicators 7 to 10) and outcome (indicators 11 to 12).

**Table 4:**
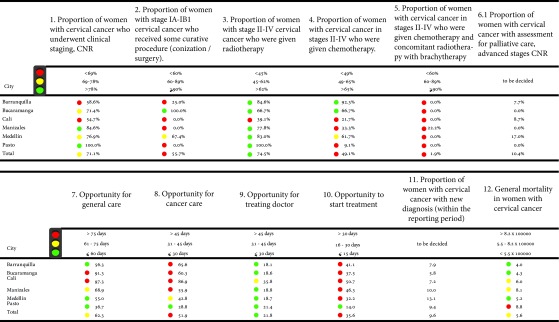
Risk management indicators in cervical cancer by city of residence

Source: High Cost Diseases Fund Database Resol 0247/14 - Cut-off date: 01 January 2016

Diagnostic measured the proportion of women who had clinical staging in the new cases, it being higher in Manizales and Pasto. On the other hand, Barranquilla and Cali had the lowest proportions. In treatment, Bucaramanga was the city with the highest proportion of women who underwent some healing procedure in stages IA-IB1, with respect to the supply of radiotherapy in stages II to IV, Pasto reported 100% of their patients receiving this therapy, the cities of Barranquilla and Bucaramanga presented the highest proportions in terms of the number of women living in these cities receiving chemotherapy.

The cities of Pasto, Medellin and Barranquilla reported times under 60 days between the time of clinical suspicion and the start of treatment. Considering the different opportunity times in a disaggregated way, Pasto presented the shortest times between the different moments of attention.

### Stomach cancer

A total of 12 indicators of the 16 considered in the consensus were measured ^16^ (Table 5). In terms of opportunity times for the diagnosis, Barranquilla and Medellin had the shortest times with 23 and 25 days, respectively. Regarding the time for the start of the first treatment after confirmation of the diagnosis Medellin was the city with the shortest time reported with 41 days, in contrast to Barranquilla, which was the city where the entities reported the highest times with 84 days. 

**Table 5:**
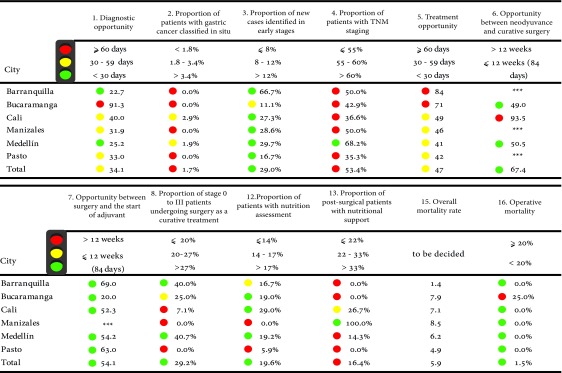
Risk management Indicators in stomach cancer by city of residence

Source: High Cost Diseases Fund Database Resol 0247/14 - Cut-off date: January 01, 2016

Medellin was the city with the highest proportion of patients with TNM staging and Barranquilla was the one with the highest proportion of staged patients in early stages. These two cities were those that in terms of treatment had the highest proportion of patients in stages 0 to III who were subjected to surgery as a curative treatment.

Bucaramanga was the only city to report cases of people with the disease who died within the first 30 days of the postoperative period.

### Colon and rectum cancer

The total numbers of indicators included in the consensus were 15 ^16^ (Table 6), of which, due to the availability of the information, 12 were measured. In terms of opportunity times, Pasto and Barranquilla had the shortest time to confirm the diagnosis, 11 and 27 days respectively; Bucaramanga presented the shortest time to start treatment. Manizales and Medellin were the cities with the highest proportion of patients with TNM staging.

**Table 6:**
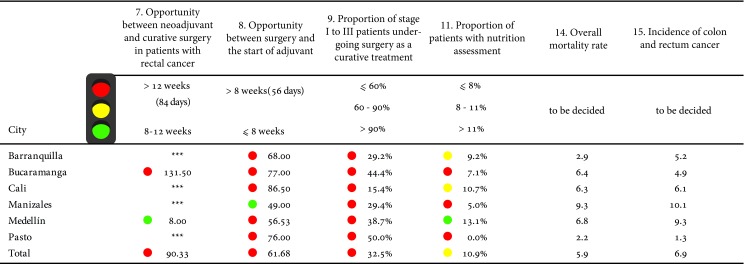
Risk management indicators in colon and rectal cancer by city of residence

Source: High Cost Diseases Fund Database Resol 0247/14 - Cut-off date: January 01, 2016

In terms of treatment, given by patients who underwent surgery with curative intent, the highest proportion of cases occurred in the city of Pasto. However, none of the cities reached the established standard.

## Discussion 

In different parts of the world, the development of indicators to evaluate the quality of cancer care has gained great importance due to the public health problem that it represents and how close to 30% of cases with this group of diseases could be avoided. Likewise, cancer is currently affecting countries especially in low and middle levels in the Human Development Index, where more than 60% of cases occur ^17^. It is urgent that governments know and monitor the actions carried out by the different factors that have influence in patients having access to quality health services and on time.

In Colombia, the measurement of indicators for the evaluation and monitoring of risk management is of great importance in order to determine whether the actions against cancer are being carried out correctly in the country and otherwise, to take effective and efficient measures to correct it.

The results for each type of cancer in the different cities are heterogeneous and show strengths and weaknesses for each of the cities and within the processes of caring for a cancer patient. The results can be approximated and be a reflection of the reality of the care process. However, there may also be weaknesses in the reporting to the High Cost Diseases Fund, with incomplete information on the part of the insurers, especially since the registry has been operating for a few years only. Likewise, the attention process is not only different according to illnesses but they also manifest themselves in a different way in each person, with certain particularities and therefore with specific times of opportunity for each one of them. However, when observing the defined standards in each of the consensus reached, a common agreement is obtained stating that for these solid characteristic neoplasms, the average time that should elapse from the moment the first doctor who has the clinical and paraclinical tools makes the decision to refer the patient for diagnostic confirmation until the first treatment is performed, there should be about 60 days in total for it to be considered good management.

It is also important to mention that the differences between cities for certain types of cancer may be due to their geographical location in the country and the availability of specialized personnel for the number of inhabitants and people with the disease in each one of them. According to figures from the National Cancer Institute in 2016 ^18^, the largest offer of oncological services in Colombia are concentrated in: Bogotá (25.1%), Antioquia (12.7%), Valle del Cauca (10.7%), Atlántico (9.1%) and, to a lesser extent, Santander (6.6%). Similarly, each capital city of these departments offers more than 88% of the cancer services available in their department ^18^.

The indicators with the best results were those related to detection in early stages, especially in gastric and colorectal cancer, these are cancers that require a specialized process for their diagnosis, different from those of the breast and cervix. However, the indicator that measures the proportion of patients with staging was low for most types of cancer, this is possibly due to the lack of reporting by insurers for this item, since staging is essential to know the extent of the disease and to plan a treatment.

With respect to treatment indicators, these usually vary by cities and by type of cancer without evidencing a clear pattern which can be explained more than by variations in the definition or standardization of treatments by regions of Colombia to differences and deficiencies in reporting, with many cases of lack of knowledge on the part of some insurers about how to report a medication or a particular treatment. Although the country has Clinical Practice Guidelines, comprehensive care routes and other protocols applicable to the entire country.

In the treatment of gastric and colon and rectum cancer, some of the most relevant indicators are those related to nutrition assessment, given that the nutritional status of patients with cancer can vary both in the initial symptoms and during the progression of the disease. It has been described in the literature that between 30 and 85% of cancer patients suffer from malnutrition, with this being a risk factor for other outcomes ^19^. However, the results show that work must be continued to reach the proposed goal, especially in the cities of Manizales and Pasto, which had the lowest patient proportions. 

In terms of opportunity times, gastric cancer was the one that showed the best results, with shorter times, especially between neoadjuvant and curative surgical management and between it and the onset of adjuvant therapy.

The measured indicators show an overview of the situation in the management of cancer by insurers in these cities, and this is considered the first step and an important input that contributes to generating information for making assertive decisions for the improvement of the quality of care for people with cancer in these cities. This articulation with the population registers for the realization of studies is crucial where causality analysis is carried out and each type of cancer is analyzed in detail. This offers the possibility of extending it to other regions in order to identify inequalities in the care process by regions but, above all, to intervene with the aim of achieving equity. 
