# Supplementary material for: Cancer risk management in Colombia, 2016
Source: Colomb Med (Cali). 2018 Mar 30;49(1):128–34. doi: 10.25100/cm.v49i1.3882 (PMC6018822; doi:10.25100/cm.v49i1.3882)
Supplement: Related texts

# Research article: Gestión del riesgo de cáncer en Colombia, 2016

## Introducción 

El cáncer es un problema de salud pública en Colombia y el mundo, que requiere de intervenciones contundentes para enfrentarlo y contenerlo, por ello se han desarrollado diversas estrategias y políticas en los diferentes países y que deben partir del conocimiento no solo clínico sino también epidemiológico del cáncer y esto atarlo a las acciones administrativas que se realizan desde el aseguramiento en salud, para ello la forma de conocer y vigilar estas acciones es a través de la medición de indicadores que den cuenta de la calidad de la atención en salud ^1^^,^^2^.

En Colombia, la información relacionada con cáncer ha tomado a lo largo de los años gran relevancia y el país trabaja en obtener información suficiente, real y veraz con el objetivo de ser analizada y direccionada a los procesos, evidenciando fortalezas y debilidades dentro del sistema de salud para la formulación de estrategias, programas y políticas que definir intervenciones correctivas. Para ello, el Ministerio de Salud y la Protección Social de Colombia (MSPS) mediante la Resolución 4496 de 2012 organiza el Sistema Nacional de Información de Cáncer (SINCan) ^3^, dentro del cual la Cuenta de Alto Costo (CAC) hace parte de esas fuentes de información, analizando datos relacionados con el aseguramiento y la prestación de servicios a los pacientes con cáncer en el país. Igualmente, el MSPS-Colombia con la Resolución 0247 de 2014, establece el reporte para el registro de los pacientes con cáncer donde la Cuenta de Alto Costo es la encargada de recolectar y consolidar la información que las entidades Administradoras de Planes de Beneficios (EAPB) incluidas las del régimen excepción y las Instituciones prestadoras de servicios de Salud públicas, privadas, mixtas y las direcciones departamentales, distritales y municipales de salud, se encuentran obligadas a reportar ^4^.

De esta forma, Colombia cuenta con un registro administrativo nacional en cáncer (RANC) desde el año 2014, con componentes clínicos, administrativos, sociodemográficos y de costos la cual desde el año 2015 es objeto de auditoria para garantizar la calidad de la información como complemento a otras fuentes de información. A partir de esta información la Cuenta de Alto Costo inicio en el año 2016 la construcción y desarrollo de procesos para estandarizar mediciones en el proceso de atención del cáncer a través de consensos basados en la evidencia para la formulación de indicadores que midieran la gestión que realizan aseguradores y prestadores en las personas con cáncer en el país.

Así mismo, otra fuente de información son los registros de cáncer de base poblacional (RCBP) en seis ciudades del país que recopilan y clasifican los casos nuevos de cáncer en residentes permanentes de Cali, Pasto, Bucaramanga, Manizales, Barranquilla y Medellín. Son miembros de la Asociación Internacional de Registros de Cáncer (IACR, por su sigla en inglés) y han divulgado la información de incidencia y supervivencia en *Cancer Incidence in Five Continentes* ^5^ y en el estudio CONCORD (6). El objetivo de este trabajo es describir los resultados de los indicadores de gestión del riesgo para cinco tipos de cáncer (estómago; colorrectal; mama; cuello uterino y próstata), en la población que se encuentra afiliada al Sistema General de Seguridad Social en Salud en Colombia (SGSSS) en las seis ciudades de Colombia que tienen RCPB.

## Materiales y Métodos 

La Cuenta de Alto Costo (CAC), es un organismo técnico del Sistema General de Seguridad Social en Salud de Colombia con la misión de Promover la gestión de riesgos, la generación de resultados en salud y la gestión de conocimiento, mediante la articulación de diferentes actores del SSGSS para disminuir la tendencia de los eventos de Alto Costo, estabilizar la variabilidad en sus manejos, asegurar la calidad técnico-científica y disminuir el impacto de la carga de enfermedad actual, mediante diversos mecanismos. Los aseguradores y proveedores de salud tienen el mandato de informar los datos de todos los pacientes con cáncer a la CAC con una periodicidad anual. La CAC es una fuente de información del SINCan encargada de integrar la información para formar el Registro Administrativo Nacional de Cáncer (RANC)

### SINCan 

Las fuentes disponibles dentro del SINCan son los registros administrativos y hospitalarios; y los estudios y encuestas poblacionales. Los datos notificados y captados desde el nivel local, territorial y nacional se integran en una bodega de datos que permite interoperabilidad de las fuentes el cual se denomina Sistema de Información Integral de la Protección Social (SISPRO) (Fig. 1). El Observatorio Nacional de Cáncer consolida la información del SISPRO, RANC y Registros Poblacionales de Cáncer para construir los indicadores para monitorear el panorama en el país, los planes de análisis y las salidas de información como herramientas necesarias para gestionar adecuadamente el conocimiento sobre mortalidad, morbilidad, acceso a servicios y acciones para el control del cáncer en Colombia. 

**Figura 1:** Sistema nacional de información de cáncer de Colombia^6^

**Definición de caso:** Personas con diagnóstico histopatológico de alguno tipo de cáncer *in situ* o invasor; personas con diagnóstico clínico de cáncer, soportado y justificado en pruebas clínicas, bioquímicas, imagenológicas o de laboratorio apropiadas en quienes, debido a alguna condición clínica, negativa a su realización o contraindicación, no fue posible realizar confirmación histopatológica hasta la fecha de corte, pero están siendo manejados como pacientes con cáncer. Para tipos de cáncer específicos, se usaron códigos de la Clasificación Internacional de Enfermedades, Décima Revisión (CIE-10) de la siguiente manera: estómago (C16); colorrectal (C18 a C21); mama (C50); cuello uterino (C53) y próstata (C61).

### Instrumento de recolección

Formulario estructurado que recolecta información en los siguientes aspectos: Identificación general de la aseguradora de salud y del usuario reportado (Preguntas (P) 1-16); Información general relacionada a diagnóstico, estadificación y objetivos del tratamiento (P 17-41); antecedentes que preceden al diagnóstico del cáncer reportado (P 42-73); información específica de cirugía (P 74-85); información específica de radioterapia (P86-105); información específica de trasplante de células madre hematopoyéticas (P 106-110); información específica de tratamiento complementario (P 111-124); situación actual del usuario a la fecha de corte (P 125-132)

### Control de calidad de la información en la CAC

La CAC realiza auditoría de la información entregada por la EAPB contra soportes clínicos; su objetivo es verificar la autenticidad del dato reportado y poder concluir certeramente la información disponible. Este proceso consta de dos grandes componentes: el primero, hace referencia a la auditoria que hace el sistema de información; a través de una malla validadora y el segundo el de verificación de la información contra historia clínica.

### Proceso de creación de los indicadores de la CAC

El desarrollo de los consensos realizados entre los diferentes actores del Sistema General de Seguridad Social en Salud en Colombia, para la identificación de los indicadores en cáncer, surgió de la necesidad de evaluar y monitorizar la gestión del riesgo en cáncer que realizan las entidades aseguradoras y los prestadores de servicios de salud, con el fin de estar alineados y contribuir con las estrategias que ha implementado el país para el control del cáncer, como lo son el plan decenal para el control del cáncer en Colombia ^1^, las guías de práctica clínica y las rutas integrales de atención de los diferentes tipos de cáncer; de esta forma generar mecanismos de control y dar un lineamiento a los profesionales en salud, entidades aseguradoras, prestadores y demás actores en la búsqueda de una mejor gestión del riesgo en salud que contribuya a disminuir la variabilidad clínica, disminuir las complicaciones de la enfermedad, mejorar la supervivencia, la calidad de vida de los pacientes y controlar el gasto en salud.

Los consensos los viene desarrollando la Cuenta de Alto Costo desde el año 2016, dándoles prioridad a los tipos de cáncer de mayor prevalencia en la sociedad colombiana, y según priorización del ministerio de salud; para ello, se tomaron los conceptos del manual metodológico de deliberación y participación del Instituto de Evaluación Tecnológica en Salud (IETS) y adapta la metodología planteada por la guía metodológica para la elaboración de guías de práctica clínica. Estas guías presentan los procesos técnicos para la formulación de la pregunta de investigación, la revisión de la literatura, la graduación de la evidencia científica y el proceso para la selección y construcción de los indicadores ^7^^,^^8^. Para la selección y construcción de los indicadores se realizó una adaptación de la metodología “The RAND/UCLA Appropriateness Method (RAM)” ^9^, que permite combinar la mejor evidencia científica disponible con el juicio colectivo de los expertos, en este caso, temáticos, metodológicos y administrativos. Para la estructuración de la pregunta se tuvo en cuenta la estrategia PICO ^10^^,^^11^, que se presentó para cada uno de los tipos de cáncer en los que se trabajó y posteriormente se diseñó un aplicativo en línea creado para el desarrollo virtual de los consensos, donde se socializaron la pregunta de investigación, los objetivos, el alcance y las limitaciones y se dispuso de un espacio de participación para los actores involucrados. 

Se realizó una búsqueda y lectura crítica de literatura y se calificó según el tipo de evidencia científica, utilizando el instrumento AGREE II ^12^ para la calificación de las guías de práctica clínica y las recomendaciones del sistema GRADE ^13^ para la calificación de los artículos de revisión. Una vez se contó con la evidencia, se procedió a la extracción de las recomendaciones y definiciones de interés, las cuales fueron revisadas y ajustadas por los participantes a través del foro virtual, de allí se generaron los posibles indicadores (el tipo de estos: proceso o resultado), el nombre, la descripción, la población objeto de aplicación (total de casos o nuevos casos) y las diferentes guías y artículos que soportaban la recomendación.

Finalmente, se definió un grupo de indicadores que fueron evaluados a través de dos votaciones virtuales y una tercera presencial, los participantes del consenso determinaron si los indicadores propuestos eran apropiados, y cumplían tres criterios esenciales: relevancia del indicador; viabilidad, entendida como la posibilidad de acceso a las fuentes de información de donde se obtendrán los datos y validez del contenido o medición que refleja lo que pretende medirse, en este caso, el indicador o indicadores que permiten evaluar la gestión del riesgo en los pacientes con cáncer. Los indicadores de gestión de riesgo para los diferentes tipos de cáncer se anexan como suplemento.

### Plan de análisis

Con la información reportada a la CAC con corte a 01 de enero de 2015, se calculó la línea de base para cada uno de los indicadores con información disponible. Según el resultado, se definieron los puntos de corte de acuerdo con los quintiles de cada indicador. Para los indicadores sin línea de base, se definieron los estándares con el apoyo de los expertos clínicos y los hallazgos de la revisión de la literatura.

Se establecieron los indicadores finales para la medición de la gestión del riesgo con el acuerdo de todos los participantes en la tercera reunión virtual y se dio por finalizado el consenso A partir de esto, se analiza cada año la información y se identifican debilidades y fortalezas del proceso de atención en cáncer. 

Para esta ocasión se describirán los resultados de los indicadores de gestión del riesgo para cinco tipos de cáncer (mama (solo en mujeres), cuello uterino, próstata, colon y recto y gástrico) en población que se encuentra afiliada al Sistema General de Seguridad Social en Salud en Colombia (SGSSS) en seis ciudades del país (Barranquilla, Bucaramanga, Cali, Manizales, Medellín y Pasto) donde operan los registros poblacionales, como complemento para el análisis y planteamiento de intervenciones para el control del cáncer. 

Los datos provienen del registro administrativo de cáncer expedido por el Ministerio de Salud y Protección Social (Resolución 0247 de 2014) y corresponden a los casos nuevos reportados (diagnosticados) entre el 02 de enero de 2015 y el primero de enero de 2016.

Se procedió con el cálculo de cada uno de los indicadores contemplados en los consensos basados en la evidencia diseñados por la Cuenta de Alto Costo, que mide la gestión del riesgo por parte de aseguradores y prestadores para los pacientes con cada uno de los tipos de cáncer anteriormente mencionados y que tienen definidos unos puntos de corte estándar con un color para cada indicador, que refleja si el resultado es bueno (verde), regular (amarillo) o malo (rojo).

Se uso el software estadístico Stata-V13 para la obtención de los datos.

## Resultados

La Tabla 1 describe el número de casos nuevos de los cánceres priorizados en el Plan Decenal para el Control de Cáncer en Colombia que fueron notificados a la CAC durante el año 2015 en las seis ciudades estudiadas

**Tabla 1:** Casos nuevos de cáncer notificados por las aseguradoras de salud a la Cuenta de Alto Costo, Colombia, 2015.

Ciudad	Mama	Próstata	Cuello uterino	Colon		Estómago		Total
	C50*	C61*	C53*	C18-C21*		C16*		
				♂	♀	♂	♀	
Cali	235	273	59	69	80	51	42	809
Pasto	17	14	12	5	1	13	4	66
Bucaramanga	47	19	8	13	15	11	10	123
Manizales	58	29	15	13	27	20	8	170
Barranquilla	132	90	35	28	37	7	5	334
Medellín	498	250	134	103	134	80	71	1,270
Total	987	675	263	231	294	182	140	2,772

Fuente: Base de datos, Cuenta de Alto Costo, *CIE-10

### Cáncer de mama

El total de indicadores contemplados en el consenso fueron 21 ^14^ (Tabla 2), sin embargo fueron medidos 19 por la disponibilidad de la información en el registro, lo cuales se dividen en cuatro grandes grupos de indicadores: de diagnóstico y estadificación (indicadores 1 a 7), de tratamiento (indicadores 8 a 12), de oportunidad (indicadores 13 a 17) y de resultado (indicadores 18 a 19). Puede observarse que la ciudad de Cali tuvo la mayor proporción de pacientes con cáncer de mama que fueron diagnosticadas en estadios in situ o tempranos, mientras que Barranquilla y Pasto presentaron las menores proporciones. Con relación a los indicadores de estadificación, ninguno de los seis municipios obtuvo el estándar definido para considerar el resultado como óptimo, sin embargo, Medellín, para los casos nuevos, es aquel que mayor proporción de casos estadificados registra. Los indicadores relacionados con la realización de pruebas diagnósticas mostraron una baja proporción de mujeres con cáncer de mama y que contaran con resultados de las pruebas hormonales, en cuanto a resultados de la prueba HER2 la proporción aumentó en las 6 ciudades, logrando Bucaramanga el valor considerado como óptimo. 

**Tabla 2:** Indicadores de gestión del riesgo en cáncer de mama por ciudad de residencia

Fuente: Cuenta de Alto Costo. Base de datos Resol 0247 /14 - Fecha de corte: 01 enero 2016

En el ámbito del tratamiento como radioterapia, terapia antiHer2 o administración de bloqueo hormonal, los resultados se ubicaron en los rangos considerados como malos o regulares (semáforo rojo y amarillo respectivamente) en la mayoría de los municipios seleccionados de acuerdo con el estándar de medición del consenso, Cali y Medellín presentaron la mayor proporción de pacientes con carcinoma in situ a quienes se les realizó cirugía conservadora de la mama.

En el tercer grupo de indicadores, relacionado con los tiempos de oportunidad, ninguna de las ciudades analizadas presentó un nivel considero óptimo de acuerdo con los estándares establecidos, los tiempos para la realización del diagnóstico, la atención médica y el inicio de tratamiento presentaron tiempos prolongados, por encima de los 60 días para una atención general, es decir desde la consulta por presencia de síntomas asociados al cáncer hasta el primer tratamiento, sin embargo ciudades como Medellín y Pasto se aproximan al rango adecuado. En términos de indicadores de resultado: letalidad según estadio y mortalidad, la ciudad con mejores resultados fue Medellín.

### Cáncer de próstata

El total de indicadores desarrollados para cáncer de próstata son 9 ^15^ (Tabla 3), sin embargo 6 susceptibles de medición, debido a la ausencia de información en el registro.

Para cáncer de próstata se midieron dos tiempos de oportunidad: desde la sospecha médica por primera consulta hasta el diagnóstico y desde esta hasta el inicio de primer tratamiento, para cada uno se estableció que un tiempo menor a los 30 días era el adecuado para considerarse un buen resultado, ninguna ciudad obtuvo este resultado.

**Tabla 3:** Indicadores de gestión del riesgo en cáncer de próstata por ciudad de residencia

Fuente: Cuenta de Alto Costo. Base de datos Resol 0247 /14 - Fecha de corte: 01 enero 2016

La mayor proporción de pacientes estadificados con el sistema TNM se presentaron en las ciudades de Manizales y Medellín, y aquellas con mayor cantidad de pacientes estadificados en estadios localizados fueron Bucaramanga y Medellín.

Las ciudades de Medellín y Pasto fueron las ciudades con mayor proporción de pacientes estadificados en Gleason score, mientras que la ciudad de Manizales tuvo la menor proporción.

### Cáncer de cuello uterino

El total de indicadores medidos para cáncer de cuello uterino fueron 12 de un total de 14 del consenso ^14^ (Tabla 4), lo cuales al igual que los indicadores de cáncer de mama, se dividen en cuatro grandes grupos de indicadores: de diagnóstico (indicador 1), de tratamiento (indicadores 2 a 6), de oportunidad (indicadores 7 a 10) y de resultado (indicadores 11 a 12). 

**Tabla 4:** Indicadores de gestión del riesgo en cáncer de cuello uterino por ciudad de residencia

Fuente: Cuenta de Alto Costo. Base de datos Resol 0247 /14 - Fecha de corte: 01 enero 2016

En diagnóstico se midió la proporción de mujeres quienes contaban con estadificación clínica, en los casos nuevos, siendo más alta en Manizales y Pasto, por otra parte, Barranquilla y Cali presentaron las proporciones más bajar. En tratamiento, Bucaramanga fue la ciudad con la mayor proporción de mujeres a quienes se les realizó algún procedimiento curativo en estadios IA-IB1, con respecto al suministro de radioterapia en estadios II a IV, Pasto reporto al 100% de sus pacientes recibiendo esta terapia, las ciudades de Barranquilla y Bucaramanga presentaron las proporciones más altas en cuanto a la cantidad de mujeres residentes en estas ciudades recibiendo quimioterapia.

La ciudad de Pasto, Medellín y Barranquilla reportaron tiempos por debajo de los 60 días entre el momento que existe la sospecha clínica y el inicio de tratamiento. Considerando los diferentes tiempos de oportunidad de forma desagregada, Pasto presentó los menores tiempos entre los diferentes momentos de la atención.

### Cáncer de estómago

Fueron medidos 12 indicadores de 16 contemplados en el consenso ^16^ (Tabla 5), en términos de tiempos de oportunidad para la realización de diagnóstico, Barranquilla y Medellín presentaron los menores tiempos con 23 y 25 días respectivamente, en cuanto al tiempo para el inicio del primer tratamiento a partir de la confirmación del diagnóstico Medellín fue la ciudad con el menor tiempo reportado con 41 días frente a Barranquilla que fue aquella ciudad donde las entidades reportaron los mayores tiempos con 84 días. 

**Tabla 5:** Indicadores de gestión del riesgo en cáncer de estómago por ciudad de residencia

Fuente: Cuenta de Alto Costo. Base de datos Resol 0247 /14 - Fecha de corte: 01 enero 2016

Medellín fue la ciudad con la mayor proporción de pacientes con estadificación TNM y Barranquilla fue la que de los pacientes estadificados la mayor proporción se encontraba en estadios tempranos. Estas dos ciudades fueron aquellas que en cuanto a tratamiento presentaron la mayor proporción de pacientes en estadios 0 a III fueron sometidos a cirugía como tratamiento curativo.

Bucaramanga fue la única que reporto casos de personas con la enfermedad que fallecieron en los primeros 30 días del postoperatorio.

### Cáncer de colon y recto

El total de indicadores contemplados en el consenso fueron 15 ^16^ (Tabla 6), de los cuales por disponibilidad de la información se midieron 12. En términos de tiempos de oportunidad Pasto y Barranquilla presentaron los menores tiempos para la confirmación del diagnóstico, 11 y 27 días respectivamente; Bucaramanga fue aquel que presentó el menor tiempo para el inicio de tratamiento. Manizales y Medellín fueron las ciudades con una mayor proporción de pacientes con estadificación por TNM.

**Tabla 6:** Indicadores de gestión del riesgo en cáncer de colon y recto por ciudad de residencia

Fuente: Cuenta de Alto Costo. Base de datos Resol 0247 /14 - Fecha de corte: 01 enero 2016

En cuanto a tratamiento, dado por los pacientes que fueron sometidos a cirugía con intención curativa, la mayor proporción se casos se presentaron en la ciudad de Pasto, sin embargo, ninguna ciudad alcanzó el estándar establecido.

## Discusión

En diferentes partes del mundo el desarrollo de indicadores para evaluar la calidad de la atención en cáncer ha cobrado gran importancia debido al problema de salud pública que representa y como cerca al 30% de los casos con este grupo de enfermedades pudieron evitarse, así mismo, el cáncer está actualmente afectando especialmente a países en niveles bajo y medio en el Índice de Desarrollo Humano, donde ocurren más del 60% de los casos ^17^ y es apremiante que los gobiernos conozcan y vigilen las acciones que realizan los diferentes actores que influyen en que los pacientes accedan con calidad y a tiempo a los servicios de salud.

En Colombia, la medición de indicadores para la evaluación y monitoreo de la gestión de riesgo son de gran importancia para determinar si se están realizando las acciones contra el cáncer de manera correcta en el país, y de no ser así, tomar medidas efectivas y eficientes para corregirlo.

Los resultados para cada tipo de cáncer en las diferentes ciudades son heterogéneos y evidencian fortalezas y debilidades para cada una de las ciudades y dentro de los procesos de la atención de un paciente con cáncer; los resultados pueden aproximarse y ser un reflejo de la realidad del proceso de atención sin embargo, también pueden existir debilidades en el reporte a la Cuenta de Alto Costo, con información incompleta por parte de las aseguradoras debido especialmente a que el registro lleva pocos años de funcionamiento, así mismo el proceso de atención no solo es diferente por enfermedades sino que estas se manifiestan igualmente de forma distinta en cada persona, con determinadas particularidades y por ende con tiempos de oportunidad específicos para cada uno de ellos, sin embargo, al observar los estándares definidos en cada uno de los consensos realizados, se llega al acuerdo común que para estas neoplasias de característica sólida, el tiempo promedio que debe transcurrir desde que el primer médico que cuenta con la herramientas clínicas y paraclínicas decide tomar la decisión de remitir al paciente para confirmación de diagnóstico hasta la realización del primer tratamiento deben pasar cerca de 60 días en total para considerarse una buena gestión

De igual forma es relevante mencionar que las diferencias entre las ciudades para determinados tipos de cáncer puede deberse a su localización geográfica en el país y a la disponibilidad de personal especializado para la cantidad de habitantes y personas con la enfermedad en cada una de ellas; según cifras del Instituto Nacional de Cancerología en el 2016 (18), la mayor oferta de los servicios oncológicos en Colombia se encuentra concentrada en: Bogotá (25.1%), Antioquia (12.7%), Valle del Cauca (10.7%), Atlántico (9.1%) y en menor proporción en Santander (6.6%), así mismo cada ciudad capital de estos departamentos ofertan más del 88% de los servicios de cáncer disponibles en su departamento ^18^.

Los indicadores con mejores resultados fueron aquellos relacionados con la detección en estadios tempranos especialmente en cáncer gástrico y de colon y recto, tipos de cáncer que requieren de un proceso especializado para su diagnóstico, diferente a los de mama y cuello uterino, sin embargo, el indicador que mide la proporción de pacientes con estadificación fue baja para la mayoría de los tipos de cáncer, esto es posiblemente debido a la falta de reporte por parte de las aseguradoras para este ítem, ya que la estadificación es indispensable para conocer la extensión de la enfermedad y planificar un tratamiento.

Con respecto a los indicadores de tratamiento, estos suelen variar por ciudades y por tipo de cáncer sin evidenciar un patrón claro, lo cual puede explicarse más que a variaciones en la definición o estandarización de tratamientos por regiones de Colombia, ya que el país cuenta con Guías de Práctica Clínica, rutas de atención integral y demás protocolos aplicables para todo el país, a diferencias y deficiencias en el reporte de estos, con desconocimiento en muchos casos por parte de algunas aseguradores sobre el cómo reportar un medicamento o un tratamiento en particular. En tratamiento de cáncer gástrico y de colon y recto uno de los indicadores de gran relevancia son los relacionados con la valoración por nutrición dado que el estado nutricional de los pacientes con cáncer puede variar tanto en el cuadro inicial como durante la progresión de la enfermedad, se ha descrito en la literatura que entre el 30 y 85% de los pacientes con cáncer se encuentran en problemas nutricionales por defecto (desnutrición), siendo este un factor de riesgo para otros desenlaces ^19^, sin embargo los resultados muestran que se debe seguir trabajando para alcanzar la meta propuesta especialmente en las ciudades de Manizales y Pasto quienes tuvieron las proporciones de pacientes más bajas. 

En términos de tiempos de oportunidad, el cáncer gástrico fue aquel que evidenció los mejores resultados, con tiempos más cortos especialmente entre la neoadyuvancia y el manejo quirúrgico curativo y entre este y el inicio de adyuvancia.

Los indicadores medidos evidencian un panorama general de la situación en la gestión del cáncer por parte de las aseguradoras en estas ciudades, y se considera este el primer paso e insumo importante que contribuye a generar información para la toma de decisiones asertivas para el mejoramiento de la calidad de la atención en las personas con cáncer en estas ciudades, la articulación con los registros poblacionales para la realización de estudios donde se realicen análisis de causalidad y se analice con mayor detalle cada tipo de cáncer, y la posibilidad de extenderlo a otras regiones para identificar desigualdades en el proceso de atención por regiones pero sobre todo intervenir para alcanzar la equidad.
